# Illinois Veterinary Medical and Surgical Association

**Published:** 1901-10

**Authors:** W. A. Swain

**Affiliations:** Secretary.


### ILLINOIS VETERINARY MEDICAL AND SURGICAL ASSOCIATION.

This Association met in semi-annual session at St. Nicholas Hotel, Decatur, August 8 and 9, 1901. The meeting was called to order by the President of the Association, Dr. V. G. Hunt, of Arcola, Ill. The roll-call showed a goodly number of the members of the Association in attendance.

The opening address of President Hunt was delivered in a masterly manner, and was replete with interest to the Association. An application was presented to the Association for membership by Dr. W. J. Martin, of Kankakee, which was favorably acted upon by the Committee on Membership; Dr. Martin was introduced to the members of the Association.

Upon proper motion to that effect, the President instructed the Secretary to draft resolutions of condolence on the death of Dr. P. M. Holburg, of Atlanta, Ill., a member of the Association, and that a copy thereof be sent to the widow of the deceased, and also one be spread upon the minutes of this meeting, which resolutions are as follows:

### RESOLUTIONS ON THE DEATH OF DR. P. M. HOLBURG.

WHEREAS, By the death of Dr. P. M. Holburg the Association has been deprived of one of its brightest and most faithful members; therefore, be it

*Resolved*, That in the late Dr. P. M. Holburg we recognize a man of learning and character and an able practitioner of his profession; that we shall remember him as a man of noble impulses, of genuine and kindly sympathy with his fellow-men, of a warm heart and an honest mind;

*Resolved*, That we tender our sympathy and condolence to the bereaved wife and family of the deceased in their hour of affliction;

*Resolved further*, That a copy of these resolutions be sent to the family of the deceased and a copy be spread upon the minutes of this meeting.

The Secretary was also instructed to prepare resolutions of sympathy and forward the same to Dr. N. P. Whitmore, of Gardner, on the death of his baby girl, who died July 30, 1901.

The resolutions are herewith submitted.

WHEREAS, Providence has seen fit to remove from the family circle the beloved child of Dr. and Mrs. N. P. Whitmore; and

WHEREAS, The Association sympathizes with them in their sorrow and affliction; therefore, be it

*Resolved*, That this Association tender to Dr. N. P. Whitmore and his family our heartfelt sympathy, and that a copy thereof be spread upon the minutes of this meeting.

The Committee on Membership reported on the application of Dr. A. D. Balsley for membership in the Association, and reported the applicant, for lack of evidence, not sufficiently qualified to become a member, and recommend that the application be deferred until the next meeting.

A very excellent paper was read by Dr. W. J. Martin, of Kankakee, on the subject of "Fistula," which brought out some very interesting as well as instructive points with reference to this disease; he was responded to by Drs. V. G. Hunt and S. H. Swain.

Under the "Reports of Cases" many interesting subjects were introduced and discussed in an able and intelligent manner by Drs. W. J. Martin and S. H. Swain.

An able and instructive paper by Dr. S. D. Brown, on "Laminitis," was well received, and was responded to by Drs. S. H. Swain and John Osborne. (Will appear later in the JOURNAL.)

The second day's session of the Association was called to order by President Hunt. Much discussion was indulged in over an able article which was read by Dr. S. H. Swain, on the subject of "Osteoporosis." Much that was both interesting and instructive was said upon the subject. The Association was honored with the presence of Dr. W. L. Williams, of Cornell University, who contributed some valuable points on the subject of osteoporosis.

The subject of "Anthrax" was well calculated to bring forth very interesting discussions, and a paper on the subject by President Hunt was well prepared and very favorably received; responded to by Drs. W. J. Martin and John Osborne.

On motion, the date and location of the next meeting was fixed for January 9 and 10, 1902, at Decatur, Ill.

W. A. SWAIN,
Secretary.

